# Seismic performance of steel-reinforced concrete-filled rectangular steel tubes after exposure to non-uniform fire

**DOI:** 10.1038/s41598-023-28517-z

**Published:** 2023-01-24

**Authors:** Yi Han, Yan-Hong Bao

**Affiliations:** 1 College of Water Conservancy and Hydropower Engineering, Gansu Agricultural University, Lanzhou, 730070 China; 2 Key Laboratory of Disaster Prevention and Mitigation in Civil Engineering of Gansu Province, Lanzhou University of Technology, Lanzhou, 730050 China; 3 School of Civil Engineering, Qinghai University, Qinghai, 810016 China

**Keywords:** Civil engineering, Natural hazards

## Abstract

In actual engineering, non-uniform fire boundary circumstances including single-sided fire, neighboring or related two-sided fire, and three-sided fire, are created due to the varying placements of the columns. In this paper, the seismic performance of SRCFST members subjected to non-uniform fire was investigated by the method of finite element simulation. First of all, the *P-*Δ curve, ductility coefficient, stiffness, and energy dissipation of the members following non-uniform fire were investigated. As the number of fire surfaces decreases, the maximum overfire temperature at the center of the section decreases, damage decreases, stiffness degradation decreases, and energy dissipation capacity increases. Next, the load distribution of each component in the SRCFST member was calculated using a three-sided fire as an example, the results show that steel tubes play the most dominant role in the seismic performance after fire, followed by steel sections and concrete the least. Last, a parametric study of the key variables influencing the ductility coefficient was carried out.

## Introduction

**Figure 1:**
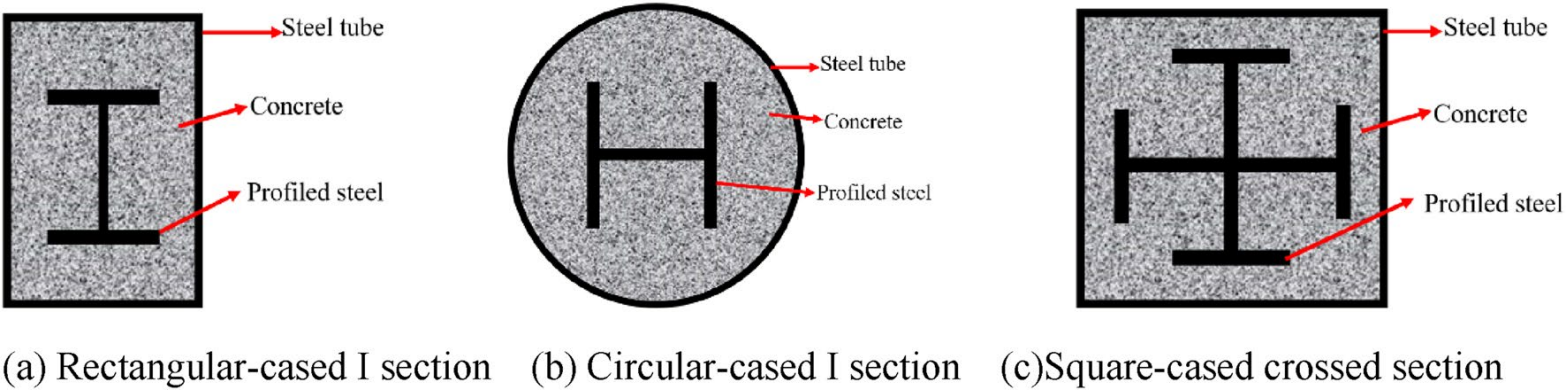
Cross-sectional shapes of SRCFST.

Steel-reinforced concrete-filled steel tubes (SRCFST) are significantly likely to be used in engineering because of their exceptional mechanical qualities. The typical cross-sectional forms are shown in Fig. 1. To improve the design method of this type of member and promote its application, scholars have conducted extensive research on the mechanical properties of SRCFST columns at room temperature. Axially compressed SRCFST members were the subject of an experimental investigation by Wang et al.^1–4^, which revealed that the steel bones could significantly increase the ductility and bearing capacity of the columns. Xu et al.^5^ performed a finite element analysis on SRCFST axial compression short columns. Based on the ultimate equilibrium theory, Ding et al.^6^ developed an equation of carrying ability while axially pressing short SRCFST columns. Zhu et al.^7,8^ created a condensed formula for the actual length to slenderness ratio and the elastoplastic bearing capacity of SRCFST axial compression long columns based on the theoretical tangential modulus approach. Unidirectional bias pressure test on SRCFST columns, Wang et al.^9^ examined the force mechanism and damage morphology. Self-compacting high-strength concrete with internal steel sections was the subject of an eccentricity test investigation^10^, which revealed that the eccentricity was the element impacting the load-bearing ability of these components. A prediction model for the ability to carry loads of SRCFST was put forward^11^, after they carried out numerical calculations on the management and sustainability of SRCFST within offset loading and found that the model in Eurocode 4 significantly underestimated the ability to bear a load of this type of member. In a finite element examination of the bending behavior of the SRCFST, Wang et al.^12^ discovered that the internally fitting profiled steel prevented the positive axis from migrating and the growth of bending cracks in the concrete. Zhao et al.^13^ created a measurement method for steel-reinforced high-strength concrete-filled steel tubes of compression-formed components. Subsequently, the mechanical characteristics of internally matched steel and steel pipe concrete columns exposed to shear^14^ and torsion^15^ have been examined consecutively. Wang et al.^16,17^ used test procedures and numerical computations to explore the mechanical characteristics of SRCFST exposure to complex loads of compression-torsion and compression-bending-shear in addition to the primary stresses on the members. Caused by the addition of profiled steel, the stiffness, peak load, and deformation performance of SRCFST members were shown to be better than those of conventional CFST columns by Xu et al.^18^ in their study of the hysteresis performance of such members. According to the investigation of Xian et al.^19,20^, the material has outstanding impact resistance on the dynamic response of SRCFST columns under horizontal impact loading by section, impact velocity, and impact direction, the material exhibits excellent impact resistance. Recent years have also seen an increase in the number of study findings on the fire resistance and fire-resistant design of such components. A finite element study of the fire resistance of SRCFST elements under non-uniform fire and during the whole fire process was carried out by Han et al.^21–23^. Meng et al.^24,25^ performed an experimental investigation on the fire resistance of this sort of component. The residual bearing capacity of SRCFST was also numerically calculated^26^ after an ISO-834 standard fire, and they also proposed a formula for predicting the residual strength index of square internal matched section steel and steel tube concrete columns under various fire exposure techniques. The post-fire seismic performance of SRCFST was researched by Han et al.^27^, and they discovered the SRCFST members performed better seismically than regular CFST members subjected to fire.

**Figure 2:**
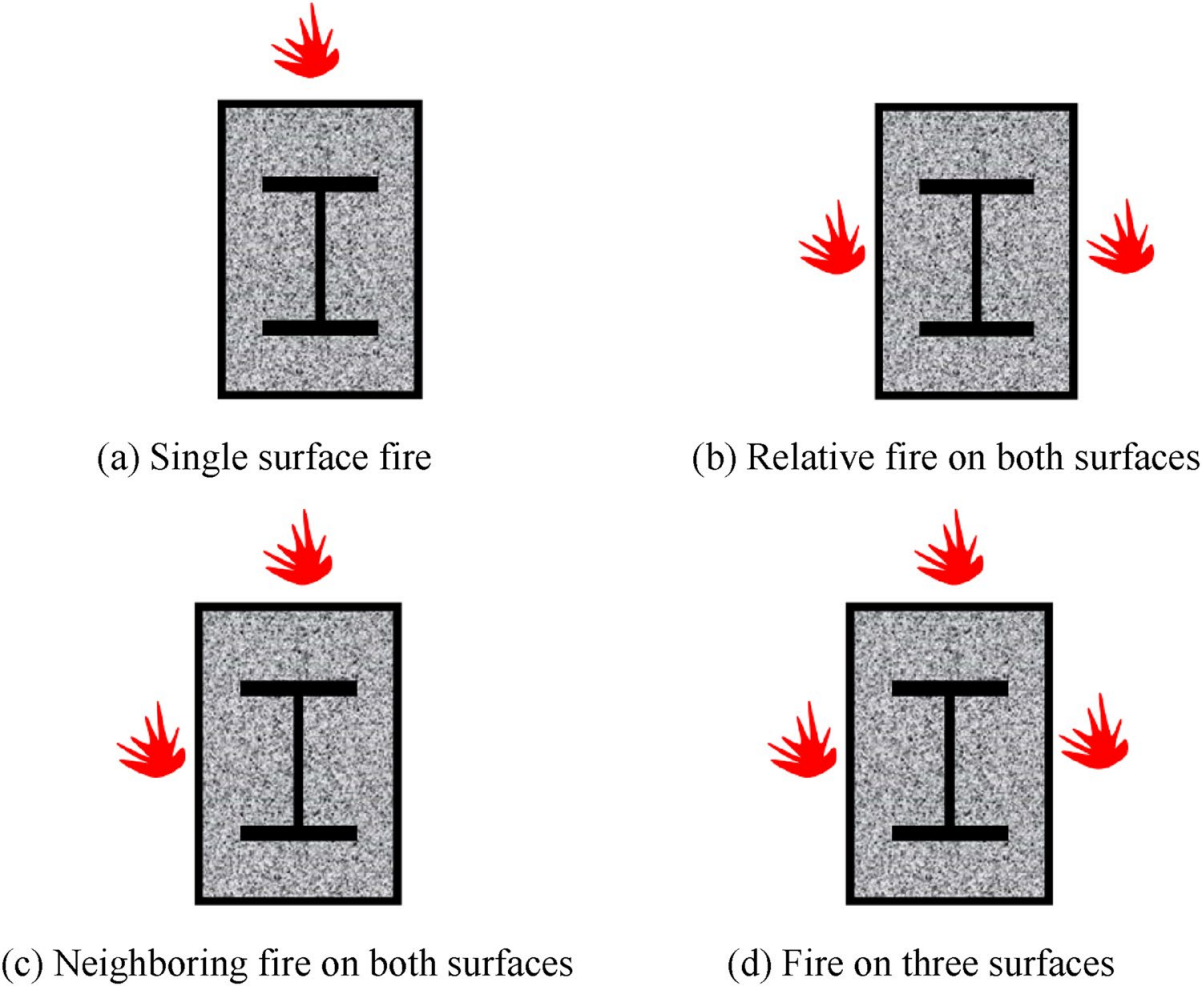
Schematic diagram of non-uniform fire conditions.

In actual engineering, owing to the varied placements of the columns, single-sided fire, relative or nearby two-sided fire, and three-sided fire will be generated, and other non-uniform fire boundary circumstances, as shown in Fig. 2. The building structure that has not collapsed after fire has to be strengthened and repaired, and if it requires seismic protection, attention should also be paid to whether its seismic performance matches those criteria.

As a result, it is essential to research the seismic performance of steel-reinforced concrete-filled steel tube columns after different fire boundary conditions, as non-uniform fire is a typical kind of fire in engineering. In this research, the finite element simulation method was used to analyze the seismic performance of SRCFST components exposed to non-uniform fire by ABAQUS. Firstly, the temperature field during the non-uniform fire was analyzed. Secondly, the hysteresis curves, skeleton lines, ductility coefficients, stiffness, energy dissipation, and other seismic indices of this kind of member are computed. Finally, under the example of fire on three sides, the contribution of steel tube, profiled steel, and concrete to the post-fire seismic performance was examined, and the ductility coefficient was parametrically examined.

## Finite element modeling

### Thermal parameters and material properties

The analysis method of sequential thermal coupling is applied in this research to first create the temperature field model and then the mechanical field model. The thermal characteristics of steel and concrete have a significant impact on the accuracy of the numerical calculation results in the temperature field model. Following a thorough review of the literature, most of the researchers employ Lie^28^ suggested by the simulation of concrete and steel thermal characteristics to determine the temperature field that is closest to the test, so this article also applies the same thermal model. The concrete heats up to around 100 °C when the water evaporates and absorbs heat, which affects the temperature field, thus the formula Han^29^ reported for the corrected capacitance and specific heat of concrete at 100 °C is utilized in this investigation, this means that it is expected that the water content in the concrete is 5% and that it all evaporates at 100 °C, as shown in Eqs. (1) and (2):

1 $$\rho_{{\text{c}}}^{^{\prime}} c_{{\text{c}}}^{^{\prime}} { = }\left\{ \begin{gathered} 0.95\rho_{{\text{c}}} c_{{\text{c}}} + 0.05\rho_{{\text{w}}} c_{{\text{w}}} \quad \quad T <100\,\,^\circ {\text{C}} \hfill \\ \rho_{{\text{c}}} c_{{\text{c}}} \quad \;\;{\kern 1pt} {\kern 1pt} {\kern 1pt} {\kern 1pt} {\kern 1pt} {\kern 1pt} {\kern 1pt} {\kern 1pt} \quad \quad \quad \quad \quad \quad T \ge 100\,\,^\circ {\text{C}} \hfill \\ \end{gathered} \right.,$$

2 $$\rho_{{\text{w}}} c_{{\text{w}}} = 4.2 \times 10^{6} {\text{J}}/({\text{m}}^{3} \cdot^\circ{\text{C}}),$$

where *ρ*_c_^’^ and *c*_c_^’^ represent the volume weight and specific heat of concrete when water vapor is taken into account; ρc and cc represent the volume weight and specific heat of core concrete when water vapor is not taken into account; *ρ*_w_ and *c*_w_ represent the volume weight and specific heat of water, respectively.

A double-fold line is used to simulate the stress–strain relationship of steel after natural cooling at a high temperature, and the specific expression is Eq. (3):

3 $$\sigma { = }\left\{ \begin{gathered} \mathop E\nolimits_{s} (\mathop T\nolimits_{\max } )\varepsilon \quad \quad \quad \quad \quad \quad \quad \quad \quad \quad \quad \quad \varepsilon \le \mathop \varepsilon \nolimits_{y} (\mathop T\nolimits_{\max } ) \hfill \\ \mathop f\nolimits_{y} (\mathop T\nolimits_{\max } ) + \mathop E\nolimits_{s}^{^{\prime}} (\mathop T\nolimits_{\max } )[\varepsilon - \mathop \varepsilon \nolimits_{y} (\mathop T\nolimits_{\max } )]\quad \;\;\varepsilon > \mathop \varepsilon \nolimits_{y} (\mathop T\nolimits_{\max } ) \hfill \\ \end{gathered} \right.,$$

The following formula (4) is used to establish the yield limit after the high temperature:

4 $$\mathop f\nolimits_{y} (\mathop T\nolimits_{{\max }} ){\text{ = }}\left\{ {\begin{array}{*{20}l} {\mathop f\nolimits_{y} } \hfill & {\mathop T\nolimits_{{\max }} \le 400\,\,^\circ {\text{C}}} \hfill \\ {\mathop f\nolimits_{y} \left[ {1{\text{ + }}2.23 \times 10^{{{\text{ - }}4}} \left( {\mathop T\nolimits_{{\max }} {\text{ - }}20} \right){\text{ - 5}}{\text{.88}} \times {\text{10}}^{{{\text{ - }}7}} \left( {\mathop T\nolimits_{{\max }} {\text{ - }}20} \right)^{2} } \right]} \hfill & {\mathop T\nolimits_{{\max }} > 400\,\,^\circ {\text{C}}} \hfill \\ \end{array} } \right.,$$

where* T*_max_ is the highest temperature in history.

In the elastic phase, the modulus of elasticity is $E_{{{\text{sp}}}} (\mathop T\nolimits_{\max } ){ = }E_{{\text{s}}} = 2.06 \times 10^{5}$ MPa, and in the strengthening phase, it is $E_{{{\text{sp}}}}^{^{\prime}} (\mathop T\nolimits_{\max } ){ = 0}{\text{.01}}E_{{\text{s}}} (\mathop T\nolimits_{\max } ) = 2.06 \times 10^{3}$ MPa.

By modifying the peak stress and peak strain of the steel tube core concrete stress–strain relationship model at ambient temperature on the basis of the equation presented by Lin^30^ in the form of equation, the stress–strain relationship of core concrete after high temperature is achieved (5).

5 $$y{\text{ = }}\left\{ {\begin{array}{*{20}l} {2x - x^{2} } \hfill & {x \le 1} \hfill \\ {\frac{x}{{\beta _{0} \left( {x - 1} \right)^{\eta } + x}}} \hfill & {x > 1} \hfill \\ \end{array} ,} \right.,$$

where $$x{ = }\frac{\varepsilon }{{\varepsilon {}_{0}}},y = \frac{\sigma }{{\sigma_{0} }},\sigma_{0} = \frac{{f_{{\text{c}}}^{^{\prime}} }}{{1 + 2.4(T_{\max } - 20)^{6} \times 10^{ - 17} }},\varepsilon_{0} = (1300 + 12.5f_{{\text{c}}}^{^{\prime}} ) \times 10^{ - 6} \times [1 + (1500T_{\max } + 5T_{\max }^{2} ) \times 10^{ - 6} ],$$

$$\eta = \left\{ \begin{gathered} 2\quad \quad \quad \;\;\;({\text{Circular section}}) \hfill \\ 1.6 + \frac{1.5}{x}\;\;\;({\text{Square and rectangular sections}}) \hfill \\ \end{gathered} \right.,$$

$$\beta_{0} = \left\{ \begin{gathered} (2.36 \times 10^{ - 5} )^{{[0.25 + (\xi - 0.5)^{7} ]}} f_{{\text{c}}}^{^{\prime}0.5} \times 0.5\quad \;({\text{Circular section}}) \hfill \\ \frac{{f_{{\text{c}}}^{^{\prime}0.1} }}{{1.2\sqrt {1 + \xi } }}\;\;\quad \quad \quad \quad \quad \quad \quad \quad \quad \;({\text{Square and rectangular sections}}) \hfill \\ \end{gathered} \right.,$$

$f_{{\text{c}}}^{^{\prime}} \;$ is the axial compressive strength of the concrete cylinder at ambient temperature, *T*_max_ is the highest temperature ever suffered, *ξ* is the constraint effect coefficient, $\xi = \frac{{A_{{\text{s}}} f_{{\text{y}}} }}{{A_{{\text{c}}} f_{{{\text{ck}}}} }}$.The slope of the tangent line of the stress–strain relationship curve past the origin is used to calculate the modulus of elasticity of core concrete after high temperature.

### Types of compartments and boundary conditions

**Figure 3:**
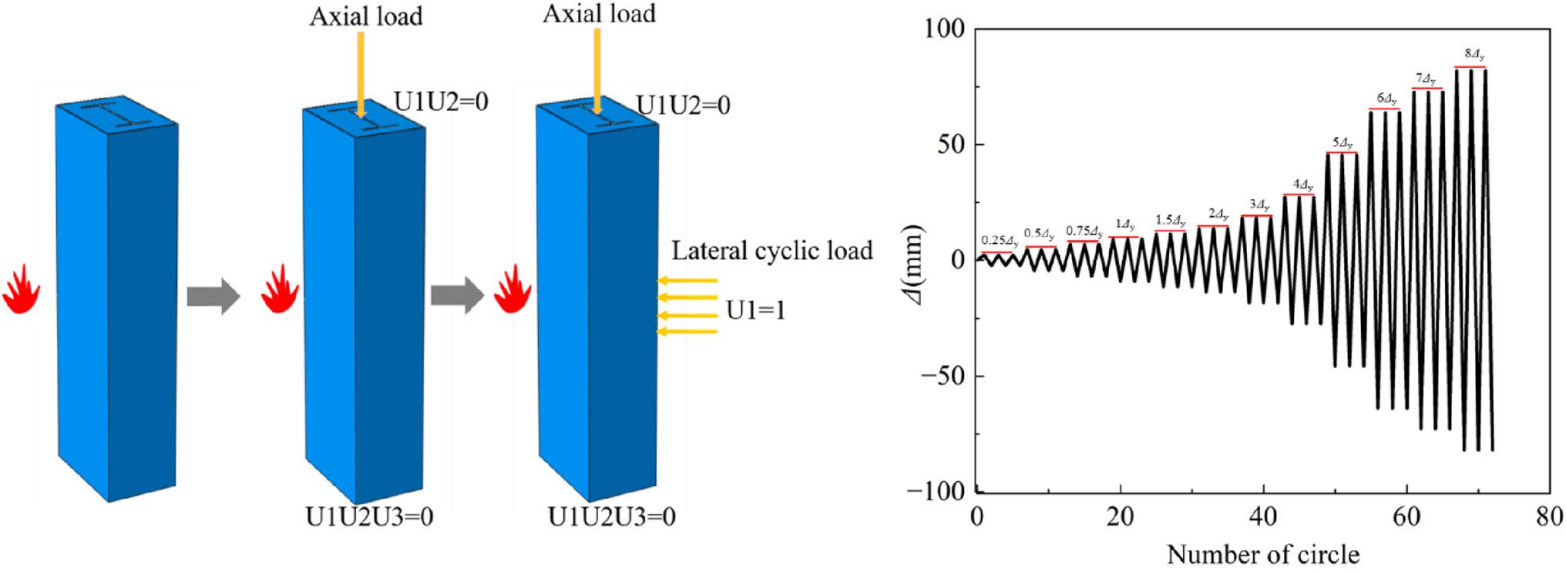
Process of loading and boundary conditions.

The SRCFST component heat transfer issues are actually non-stationary heat conduction problems without an internal heat source. The fire conditions investigated in this work are those in which thermal radiation and convection transfer heat from the outside of the structure to the column elements. The third category of boundary conditions, the ISO-834 heating curve, which accounts for the effects of convection and radiation on the component boundaries, regulates how the temperature changes during the fire heating process. For the fire surface, the heat transfer coefficient is taken as 25 W/(m∙ °C) and the integrated radiation coefficient is taken as 0.5; for the non-fire surface, the heat transfer coefficient is taken as 9 W/(m∙ °C)^31^ and the Stefan-Boltzmann constant is assumed to be 5.67×10^–8^ W/(m^3^∙K^4^) with an absolute zero of − 273 °C^29^. In the temperature field calculation model, total heat transmission is assumed, disregarding the contact heat resistance between steel and concrete, and “tie” restrictions are employed between the steel tube and concrete, concrete and profiled steel. In the mechanical field calculation model, the steel tube and concrete, profiled steel and concrete using the “surface to surface” contact, where the average direction using the hard contact, tangential operating Cullen friction model, and the friction coefficient is 0.6. Figure 3 depicts the component loading stages and boundary conditions. The loading procedure is broken down into three stages: first, heating the fire surface of the column members; second, hinged column ends with constant axial load applied at top; and third, reciprocal displacement load applied in span. Force-controlled loading, displacement-controlled loading^32^, and hybrid force–displacement controlled loading are the three primary categories of proposed static loading experimental rules that are now in use. In this paper, displacement-controlled loading is selected, i.e. the displacement during loading is used as the control quantity, and cyclic loading is performed according to a certain displacement increase, and the target displacement amplitude is obtained by referring to JGJ/T 101–2015^33^ as 0.25Δ_y_, 0.5Δ_y_, 0.75Δ_y_, 1Δ_y_, 1.5Δ_y_, 2.0Δ_y_, 3.0Δ_y_, 4.0Δ_y_, 5.0Δ_y_, 6.0Δ_y_, 7.0Δ_y_, 8.0Δ_y_, Δ_y_ is the yield displacement of the column, and each stage is cycled three times respectively. Steel tube, profiled steel, concrete, and both the mechanical and thermal fields use C3D8R units. Temperature and mechanical fields consistently mesh.

### Verification of numerical calculation model

**Figure 4:**
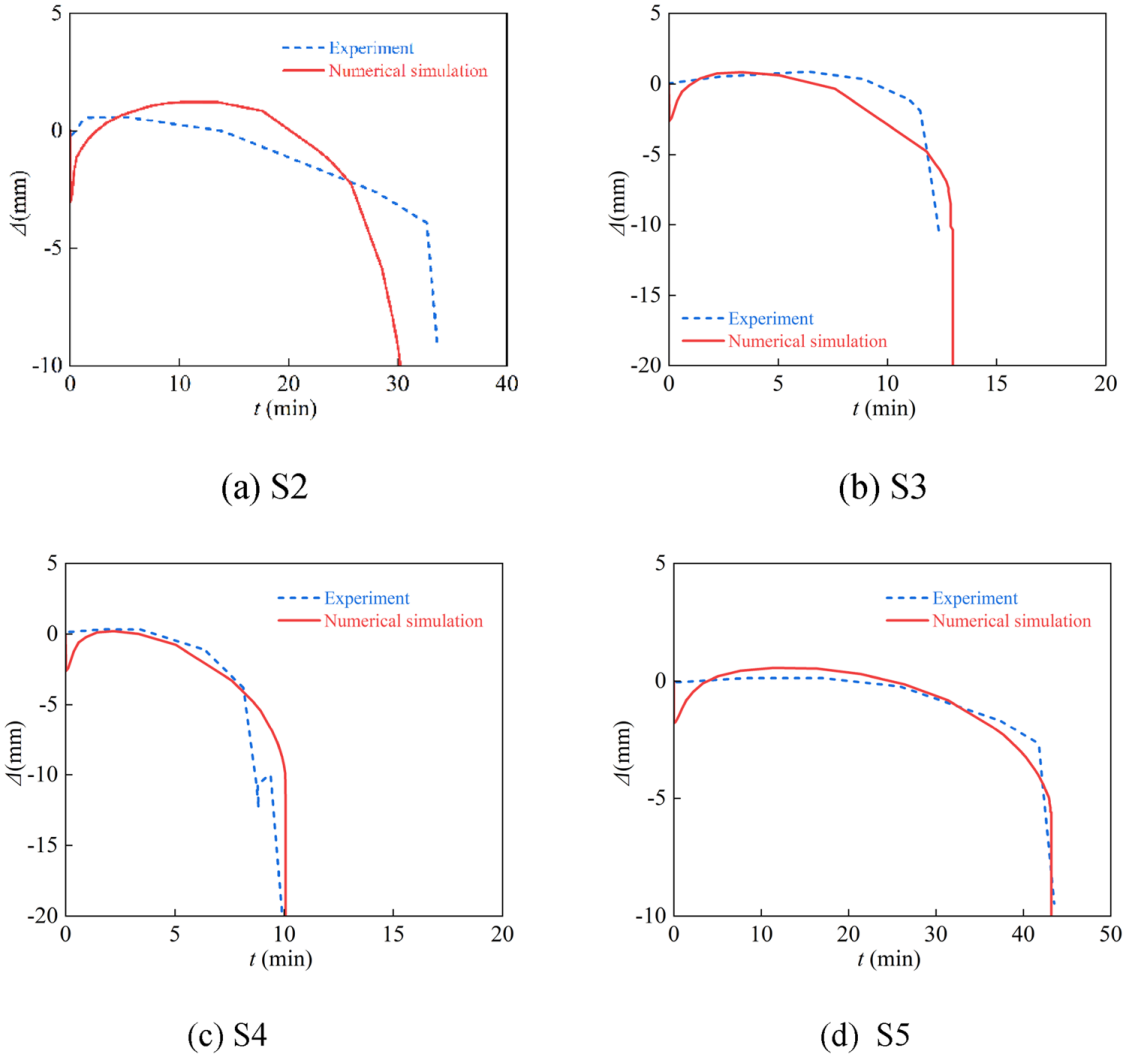
Comparison of experimental and calculated curves.

**Figure 5:**
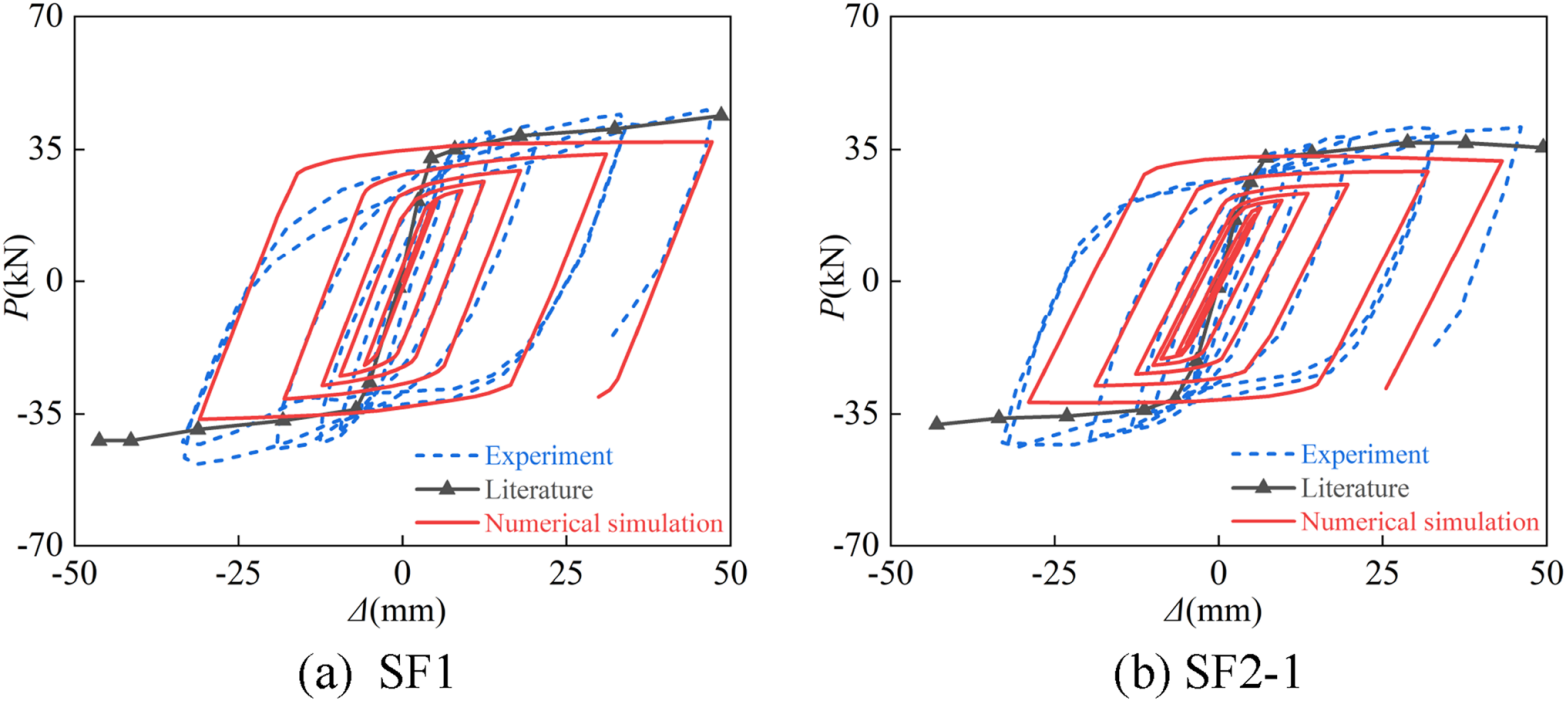
*P-*Δ hysteresis curves after exposure to fire.

Numerical calculations were done for the tests of non-uniform fire of concrete rectangular steel tube columns in the literature^34^ and the hysteresis test of concrete-filled square steel pipe columns after fire in the literature^30^. The test parameters are listed in Tables 1 and 2, and Figs. 4 and 5 display the comparison curves between the two tests.

**Table 1:** List of test specimen parameters for CFST subjected to non-uniform fire.

Specimen	Fire surface	*D* × *B* × *t*_s_ (mm)	*f*_yt_ (MPa)	*f*_cu_ (MPa)	*n*	*N*_F_ (kN)	*e* (mm)
S2	Three	300 × 300 × 5.8	361.72	59.3	0.4	2121.0	− 40
S3	Three	300 × 300 × 5.74	361.72	59.3	0.6	2312.0	− 40
S4	Three	300 × 300 × 5.74	352.30	59.3	0.6	1693.0	− 80
S5	Single	300 × 300 × 5.74	299.14	59.3	0.6	2976.6	0

**Table 2:** List of test specimen parameters for hysteretic behavior of CFST.

Section type	Specimen	*D* (*B*) × *t*_s_ (mm)	*t*_*h*_ (min)	*L* (mm)	*N*_0_ (kN)	*n*
Square	SF1	120 × 2.9	90	1500	0	0
	SF2-1	120 × 2.9	90	1500	60	0.15

The relationship curve between refractory time and axial deformation is depicted in Fig. 4. It can be shown that the numerical simulation results of the fire resistance limit under non-uniform fire are near to the test.

**Figure 6:**
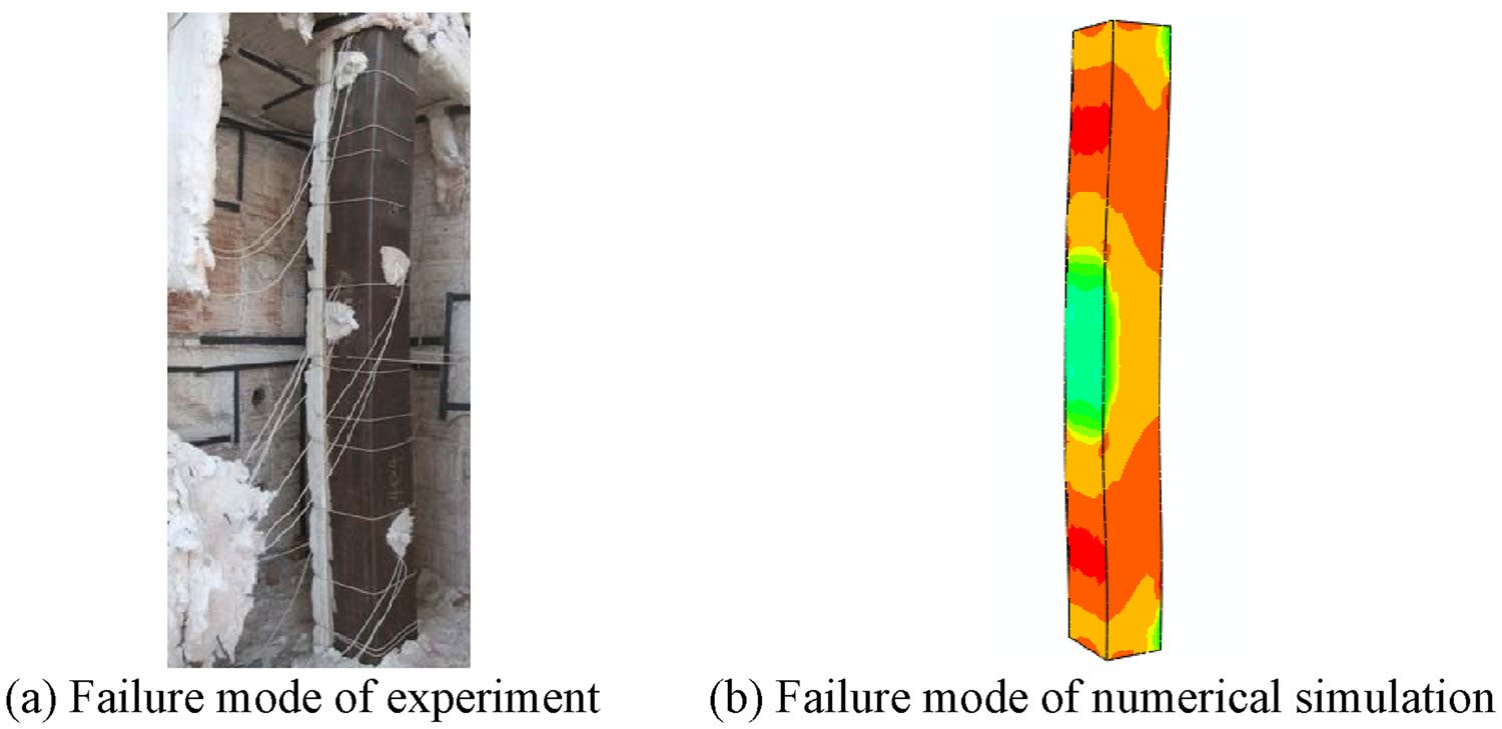
Failure modes of S3 experiment and numerical simulation.

Figure 5 shows the *P-*Δ hysteresis curve of the CFST members after fire, and it can be seen that the shape and size of the shape and size of the hysteresis loop after fire are also in excellent agreement with the experiment. Although there is some difference between the curve of the test and the curve obtained by numerical calculation, this is because the numerical calculation is idealized, while the test process will have initial errors, etc. Figure 6 shows the comparison between the numerical calculation results of the failure mode of the S3 member and the test results, which can be seen that both are in integral bending. In summary, the modeling method has a certain degree of reliability.

## Results and discussion

After different fire exposure modes were established, the standard members shown in Table 3 were designed using the modeling mentioned above, considering the requirements of GB50936-2014^35^ and JGJ138-2001^36^, in addition to the shared dimensions of the actual project. This was followed by the seismic performance analysis model of SRCFST members.

**Table 3:** Detailed information of SRCFST column.

Specimen	*D* × *B* × *t*_s_ (mm)	*t*_*h*_ (min)	*α*_*t*_	*α*_*s*_	*f*_ys_ (MPa)	*f*_yt_ (MPa)	*f*_cu_ (MPa)	*n*
SRCFST	600 × 400 × 9	90	0.08	0.05	345	345	60	0.5

*D* is the length of the rectangular section, *B* is the width of the rectangular section, *t*_s_ is the thickness of the steel tube, *t*_h_ is the heating time, *α*_*t*_ = *A*_t_/*A*_c_ (*A*_t_ is the section area of the steel tube, *A*_c_ is the section area of concrete) is steel tube ratio,* α*_*s*_ = *A*_s_/*A*_c_ (*A*_s_ is section area of profiled steel) is profiled steel ratio, *f*_ys_ is the yield strength of profiled steel, *f*_yt_ is the yield strength of steel tube, *f*_cu_ is concrete cubic compressive strength, *n* is the axial compression ratio.

### Analysis of temperature fields

**Figure 7:**
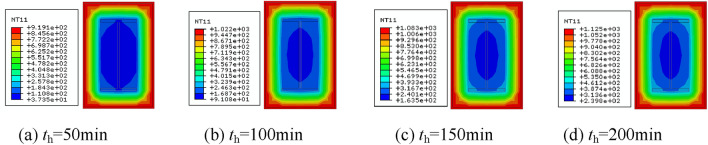
Cross-sectional temperature clouds at various stages of the fire on all four surfaces.

**Figure 8:**
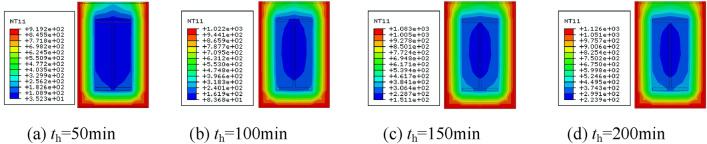
Cross-sectional temperature clouds at various stages of the fire on three surfaces.

**Figure 9:**
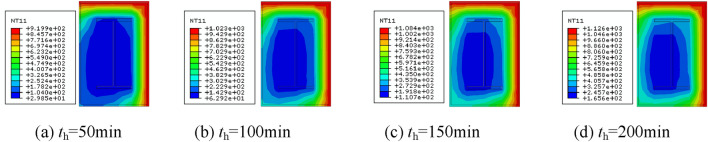
Cross-sectional temperature clouds at various stages of the fire on all two surfaces.

**Figure 10:**
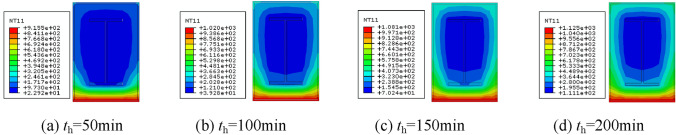
Cross-sectional temperature clouds at various stages of the fire on single surface.

The temperature clouds of the column span cross-section at different moments after different fire exposure methods are shown in Figs. 7, 8, 9 and 10. As can be observed, when the fire is uniform, the temperature field distribution is biaxially symmetric, three sides of the fire, single-sided fire, the temperature field is uniaxially symmetric, the adjacent side of the fire, the temperature field is not symmetric.

**Figure 11:**
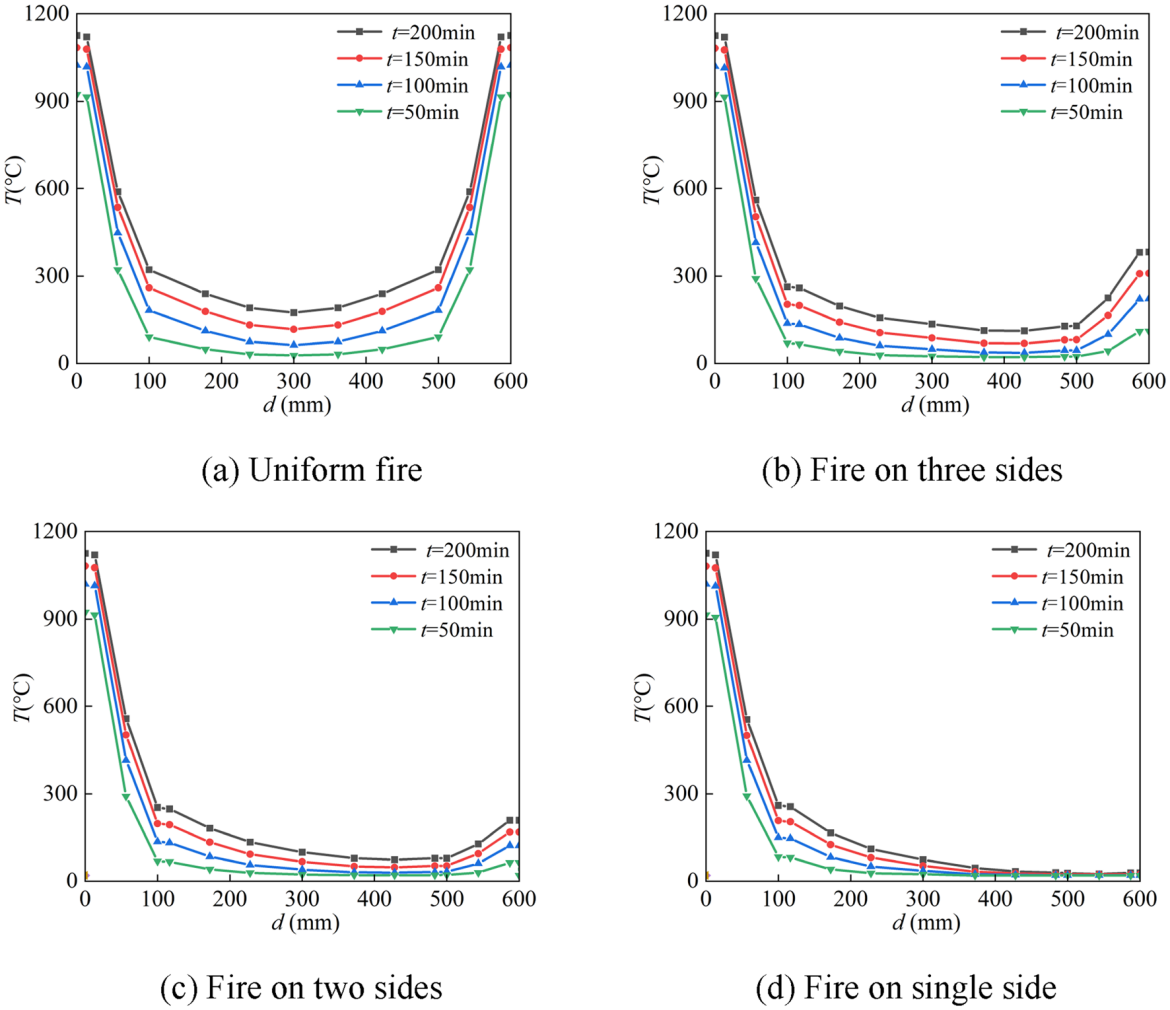
Temperature-depth curves.

Figure 11 depicts the width-temperature variation curves along the section at different times under different fire circumstances. Three sides of the fire, a larger fire surface, a hotter fire surface on the rear, and two sides of the fire after that. The temperature of the rear side is the lowest, almost at ambient temperature, when fire is applied to one side. The temperature of the backfire surface gradually rises as the heating period is extended because heat from the fire surface is continually transferred to the backfire surface. This happens even if the temperature on the fire side is very high and the temperature on the backfire side is comparatively low. The temperature field distribution is uneven due to the comparatively high temperature on the fire-receiving side and the relatively low temperature on the backfire side. This will have two consequences on the mechanical characteristics of the part, namely different eccentricity and additional deflection, resulting in non-uniform fire properties that vary from uniform fire properties.

### Analysis of the seismic performance of SRCFST members after exposure to non-uniform fire

The characteristics of the bearing capacity, ductility, and energy dissipation capacity of building structures and members are included in the seismic performance. These qualities are essential for determining how well building structures will function in the event of large earthquakes and are crucial for determining how seismically resistant they are. The findings of the calculation used in this study to determine the seismic indices for this sort of member after fire are as follows.

#### Failure modes

**Figure 12:**
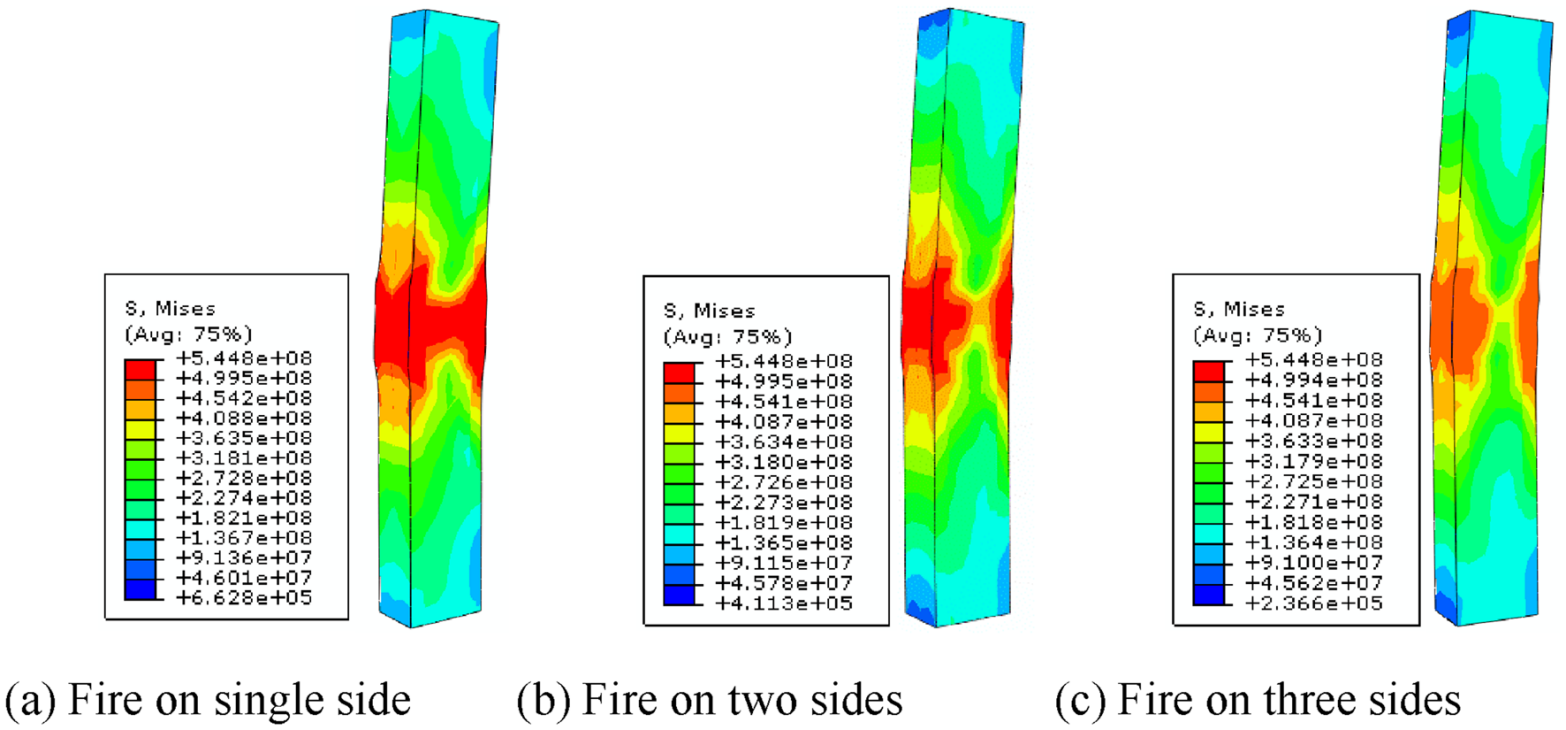
Failure mode of SRCFST members after reciprocal loading under non-uniform fire.

Figure 12 shows the failure mode of the SRCFST member under reciprocal load after non-uniform fire, and it can be seen that the failure mode of the column is the same regardless of the fire boundary condition. First, there occurs compressive buckling in the column, and then during unloading and reverse loading, the bulging section is flattened again and produces compression bulging on the other side. With increased displacement of loading and unloading, the bulging phenomena is amplified, but this type of member still has a good bearing capacity.

**Figure 13:**
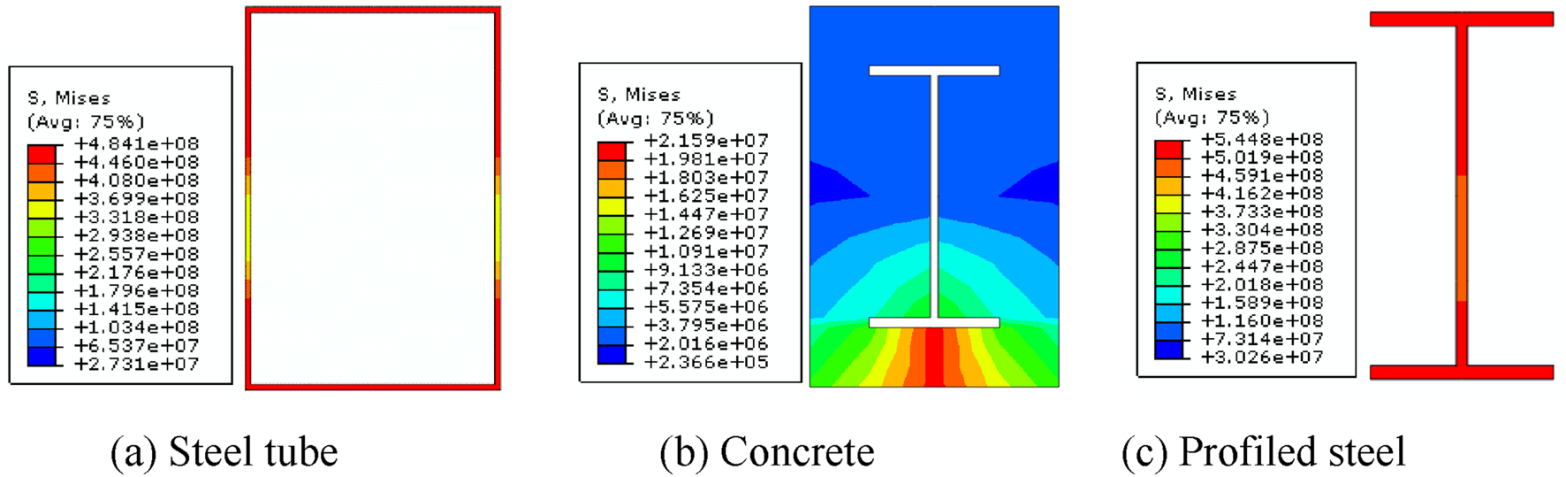
Stress cloud in the span section of SRCFST column after fire on three surfaces.

Figure 13 illustrates the stress cloud diagram of the span cross section of each component in the SRCFST member after the three-sided fire. The temperature field on the non-symmetric axis forms an asymmetry of material loss due to temperature asymmetry, thus forming an inhomogeneous material field in the cross section of the member, which causes the center of the joint force of the cross section to shift and form additional eccentric distance. The stresses in the interior profiled steel and peripheral steel tube are larger under the combined influence of temperature and reciprocating load than the stresses in the concrete, which demonstrates that the steel tube and profiled steel bear the majority of the load.

#### Hysteresis curves and skeleton lines

**Figure 14:**
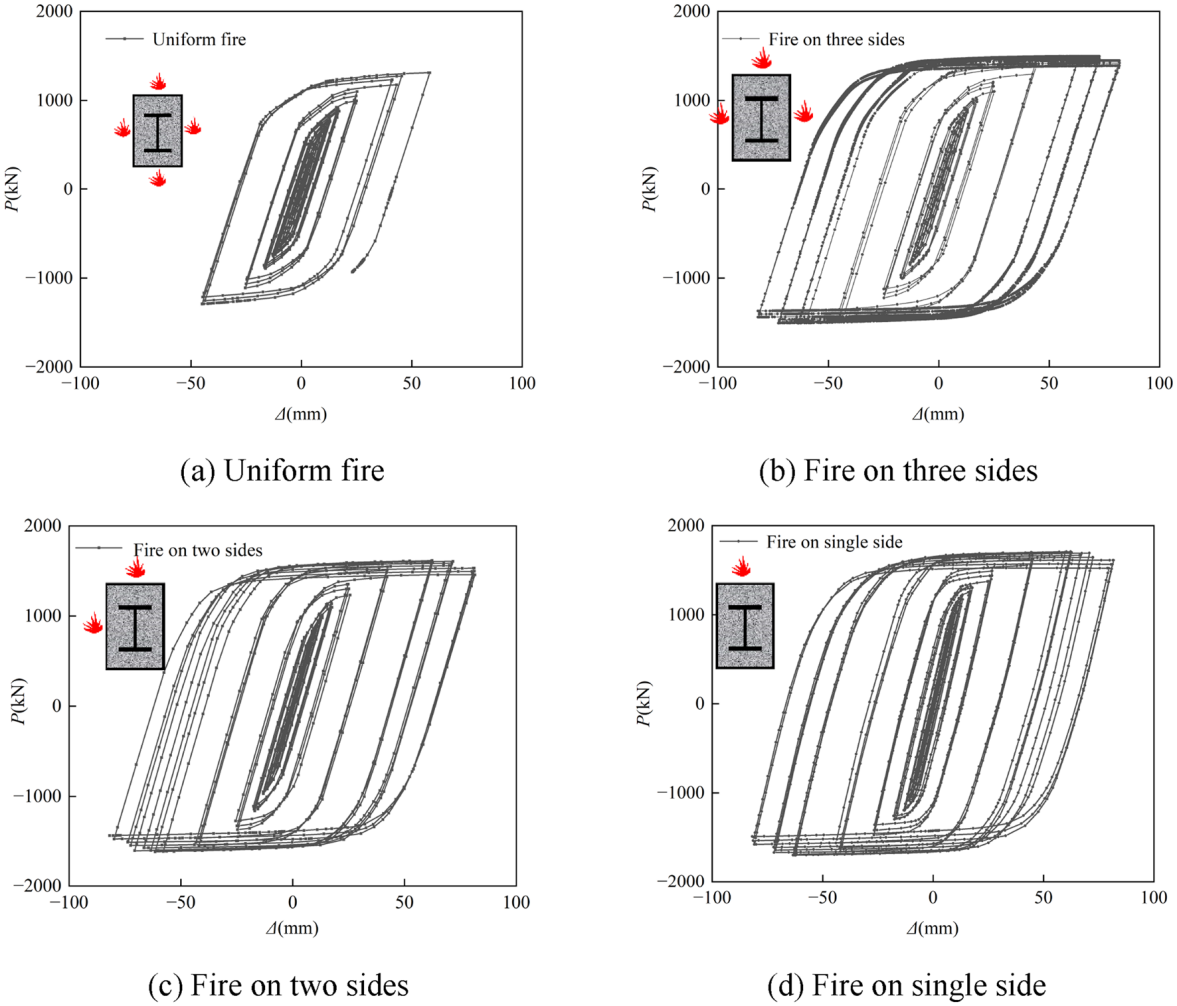
Hysteresis curves of *P-*Δ relations.

The *P-*Δ hysteresis curves of SRCFST members after uniform and non-uniform fires are shown in Fig. 14. It is clear that the members are in the elastic stage at this point since the *P-*Δ relation of the columns is near to a straight line and no evident hysteresis loop forms when the lateral deformation is minor. The area contained by the hysteresis loop progressively grows as lateral displacement rises, and all of the hysteresis curves are rather complete without any noticeable pinching issue. Because the material properties of the steel recovered after natural cooling, the contribution capacity of the outer steel tube and the inner profiled steel to the bearing capacity and ductility after fire increased. The restraining effect of the outer steel tube on the core concrete can prevent the concrete from bond damage, the profiled steel can delay or partially inhibit the generation of diagonal cracks in the core concrete, the core concrete improves the stability of the outer steel tube and the profiled steel, which can effectively prevent the strength drop caused by the buckling of the steel pipe and the profiled steel. The interaction between the steel tube, profiled steel, and core concrete is what gives these parts their significant potential to dissipate energy. In contrast, when all four sides are exposed to fire, the member sustains damage when the displacement amplitude exceeds 58.2 mm, and the hysteresis loop size is decreased resulting from the increased number of fire surfaces, high overfire temperature, and severe material degradation. In comparison to a four-sided fire, the fire surface is decreased, which also affects the historical maximum temperature, material degradation, member hysteresis loop size, and peak load of each hysteresis loop.

**Figure 15:**
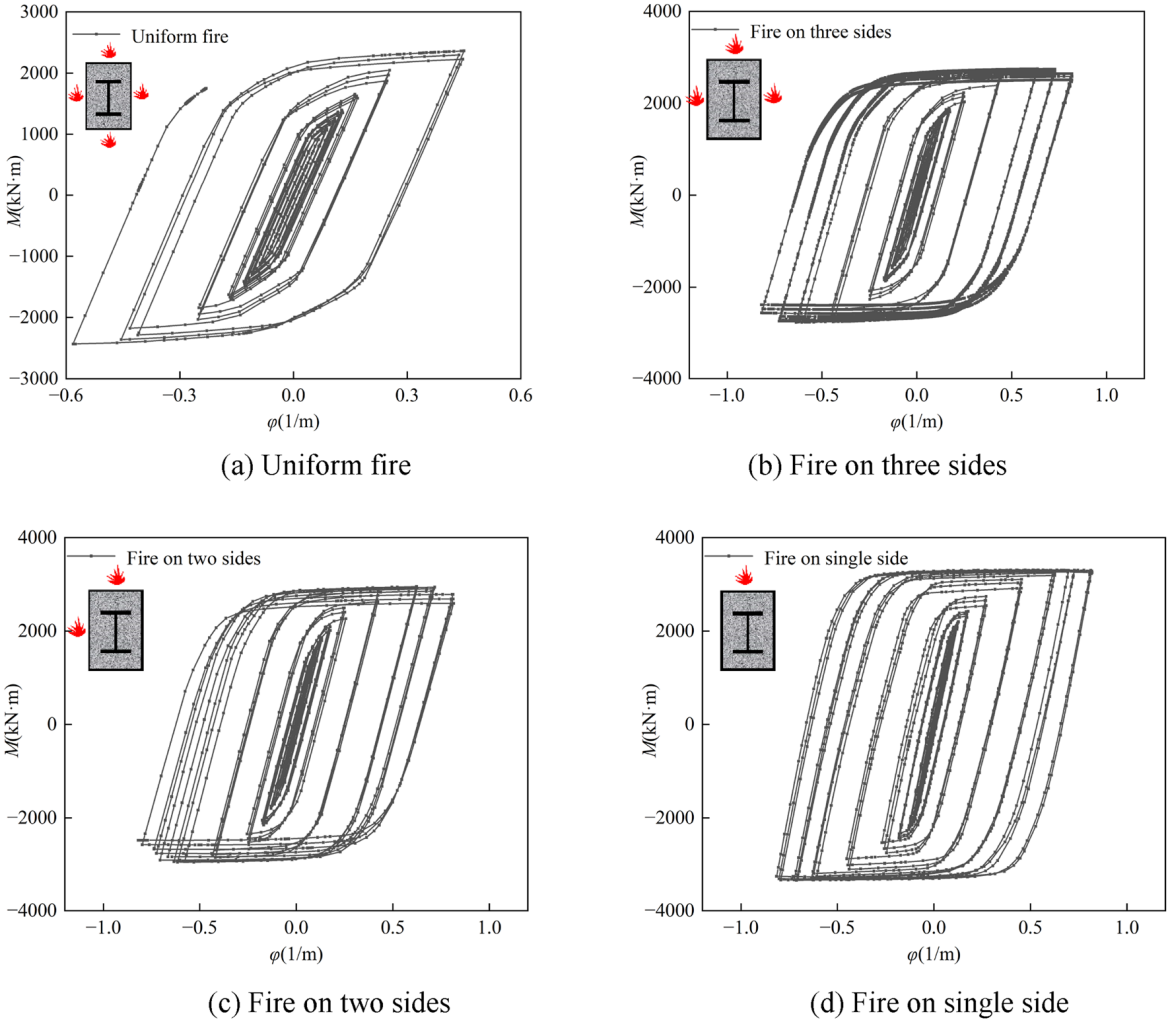
Hysteresis curves of *M-φ* relations.

Figure 15 shows the moment–curvature hysteresis curves of the spanwise sections of the SRCFST columns. It can be seen that the shapes of the *M-φ* hysteresis curves of the SRCFST members after fire are fuller, except for the four sides of the fire. The *P*-Δ hysteresis curve is comparable to the distinctive law of *M-φ* of SRCFST members under various fire situations; when the number of fire surfaces decreases, the hysteresis loop area grows, and the *M-φ* hysteresis loop area is applied to one side, it is at its greatest and fullest. It can be further concluded from the *M-φ* hysteresis curve that SRCFST columns have excellent seismic performance exposure to fire.

**Figure 16:**
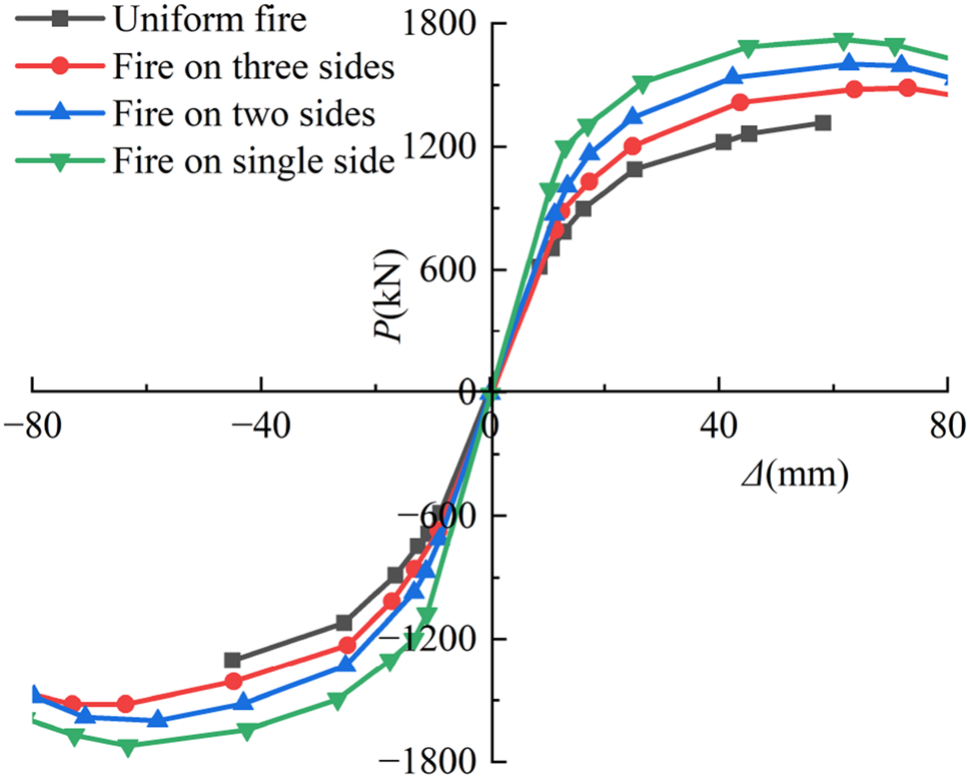
Skeleton curves of P-Δ relations.

Figure 16 depicts the *P-*Δ skeleton line of a typical SRCFST member after taking into account various fire events. The variation law of the member skeleton line is essentially the same in all four operating circumstances. The ultimate load capacity of the part decreases as the fire surface increases. The ultimate bearing capacity of a four-sided fire is 13.54% lower than that of a three-sided fire, and the ultimate bearing capacity of a three-sided fire is 5.03% lower than that of a two-sided fire, while the ultimate bearing capacity of a two-sided fire is 7% lower than that of one-sided fire. As can be observed, the skeleton line is less affected by temperature fluctuations as the number of fire surfaces reduces, and the lateral reciprocating load becomes more critical as a regulating factor. Additionally, it can be observed that non-uniform firing causes the skeleton line to descend at a somewhat higher angle. This is because, under these three fire circumstances, the section of specimen strength center is offset to the unfired surface, and the line of the horizontal force action does not pass through the section strength center, resulting in eccentricity.

#### Stiffness degradation

**Figure 17:**
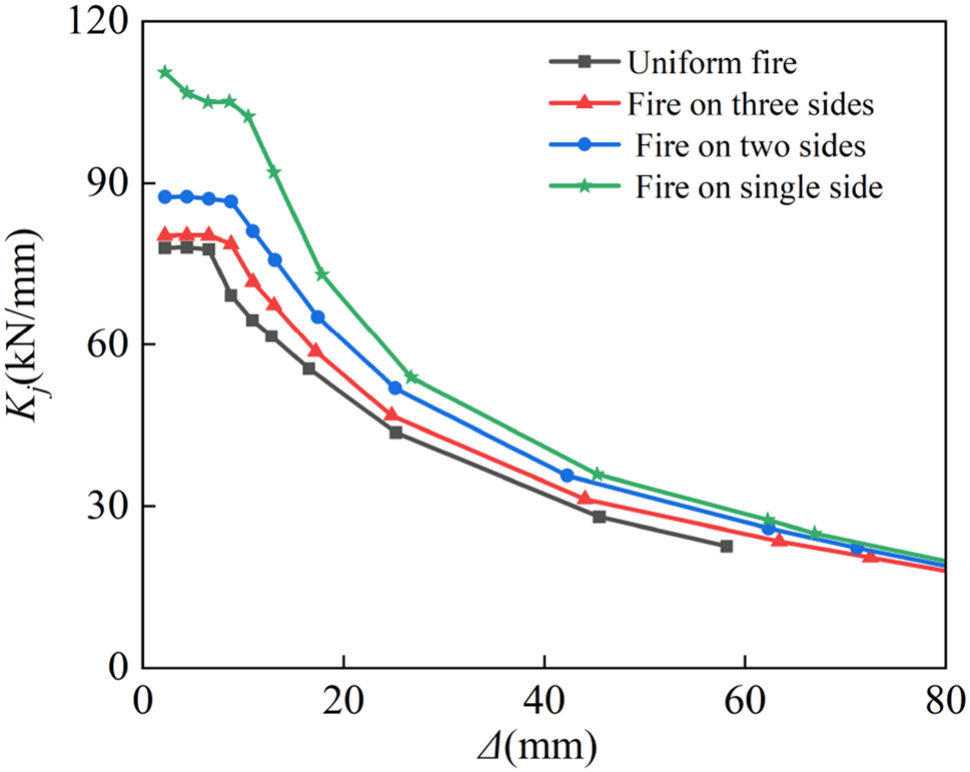
Stiffness degradation.

The cut-line stiffness, which is determined using the following formula (6), is used to represent the stiffness of specimens^36^:

6 $$K_{j} = \frac{{\left| { + \left. {P_{j} } \right| + \left| { - \left. {P_{j} } \right|} \right.} \right.}}{{\left| { + \left. {\Delta_{j} } \right| + \left| { - \left. {\Delta_{j} } \right|} \right.} \right.}},$$

where *P*_j_ is the positive and negative peak point load value during the first cycle of level *j*, and Δ_j_ is the corresponding displacement. The calculation results are shown in Fig. 17. It is evident that regardless of the fire circumstances, the combined effects of fire and cyclical stress cause the stiffness to diminish steadily. The contribution of temperature action to the stiffness degradation of the member is more sensitive at the beginning of loading. Compared to four-sided flames, the stiffness rose when Δ = 2.2 mm by 2.84%, 12.03%, and 41.51% for three-sided, adjacent-sided, and one-sided fires, respectively. Consequently, the stiffness increases as the number of fire surfaces decrease. For three-sided, adjacent-sided, and one-sided flames, respectively, the stiffness is enhanced by 7.31%, 18.92%, and 26.12% when Δ = 25 mm compared to four-sided fires. The difference in stiffness of the members with various fire types rapidly diminishes as the reciprocating force rises, the impact of temperature on the columns’ stiffness declines, and the lateral load exerts control.

#### Ductility

The deformation capacity of a component is its ductility, which is often represented by the ductility coefficient, which is described this way^36–39^:

7 $$\mu { = }\frac{{\Delta_{{\text{u}}} }}{{\Delta_{y} }},$$

where Δ_y_ denotes the yield displacement and *Δ*_u_ the ultimate displacement. The technique suggested by Park et al.^40^ was used to compute the yield displacement, and the calculation findings are displayed in Table 4. Since it can be seen, the yield load and peak load of the members are much more significant after various non-uniform fire techniques than after uniform fire, and the increased value rises as the number of fire surfaces of the specimens decreased. The yield loads of the specimens following fire on three sides, fire on neighboring sides, and fire on one side rose by 12.59%, 21.62%, and 28.71%, respectively, while the peak loads increased by 14.84%, 22.29%, and 31.52%, respectively, as compared to the evenly burnt members. This is mainly because fires that reach high temperatures drastically degrade the mechanical qualities of steel and concrete. Additionally, as the number of fire surfaces grows, the cross-section of the component overfire temperature field expands, leading to more severe post-disaster damage. The ductility coefficient is most significant when the fire is used to one side, lowest when used to four sides, and the difference between the ductility coefficients when the fire is applied to neighboring sides and three sides is not very significant. Three-sided fire, adjacent-sided fire, and single-sided fire all had higher ductility coefficients than uniform fire, increasing by 43.4%, 52.6%, and 84.2%, respectively. In the case of non-uniform fire, the number of fire surfaces causes an increase in the yield displacement of the member, a relatively small change in the ultimate displacement, and a drop in the ductility coefficient.

**Table 4:** The results of ductility.

Receiving fire mode	Direction	Yield load *P*_y_/kN	Yield displacement Δ_y_/mm	Maximum load *P*_max_/kN	Maximum displacement Δ_max_/mm	Failure displacement Δ_u_/mm	Ductility *μ*	
Uniform fire	+	1107.1	27.2	1314.73	58.2	58.2	2.1	1.9
	−	1131.2	26.7	1303.5	45.0	45.0	1.7	
Fire on three sides	+	1248.1	29.0	1486.2	82.1	82.1	2.8	2.8
	−	1272.1	29.6	1520.6	82.1	82.1	2.8	
Fire on two sides	+	1361.4	26.8	1601.2	81.2	81.2	3.0	2.9
	−	1360.8	28.1	1600.7	79.7	79.7	2.8	
Fire on single side	+	1441.3	23.4	1721.7	82.0	82.0	3.5	3.5
	−	1439.7	24.0	1721.7	81.2	81.2	3.4	

#### Energy dissipation

**Figure 18:**
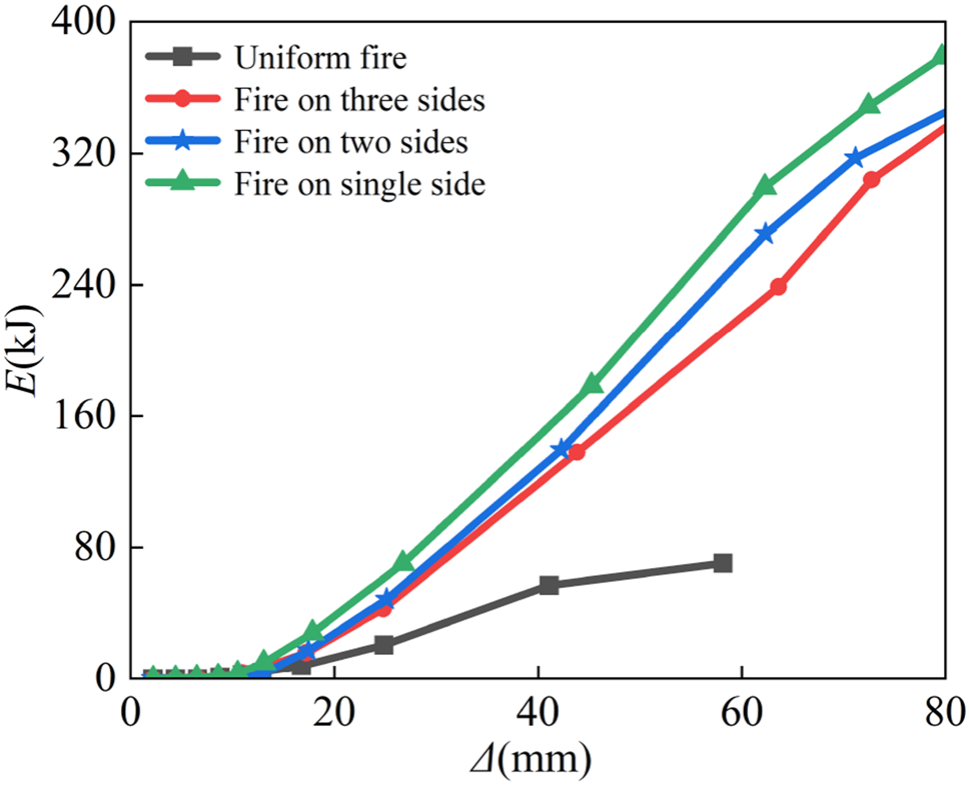
Energy dissipation.

A structure is exposed to an earthquake, which introduces energy into the structure and causes it to absorb and release energy continuously. The capacity of the members to dissipate energy determines how well the system performs seismically when it transitions into the elastic–plastic condition. The ability of a structural member to dissipate energy is determined by the area encompassed by its load-deformation hysteresis curve, and the accumulation of this area indicates the structure’s elastic–plastic energy dissipation in terms of its magnitude. Figure 18 shows the curve of dissipated energy *E* versus lateral displacement Δ*.* It is evident that as the number of fire surfaces increases, each specimen cumulative hysteresis energy consumption decreases. However, hysteresis energy consumption is minimized when the fire is uniform, as this causes the column section to experience high overfire temperatures, severe material deterioration, and premature member damage. Additionally, it is clear that when the lateral displacement is minor, the member is essentially in an elastic condition and that the region encircled by the hysteresis loop is tiny, which results in low energy consumption. The member enters the plastic phase when the lateral displacement rises, the area of the hysteresis loop expands, and the energy dissipation increases.

#### Load distribution of each component

**Figure 19:**
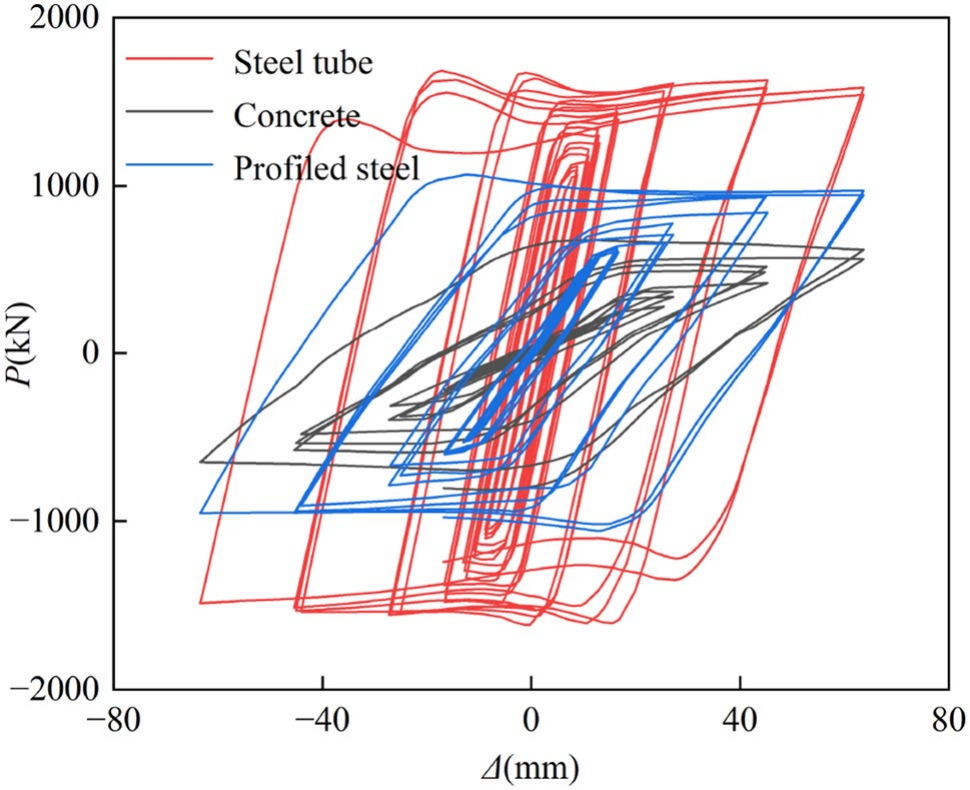
Hysteresis curves of *P-*Δ relations.

**Figure 20:**
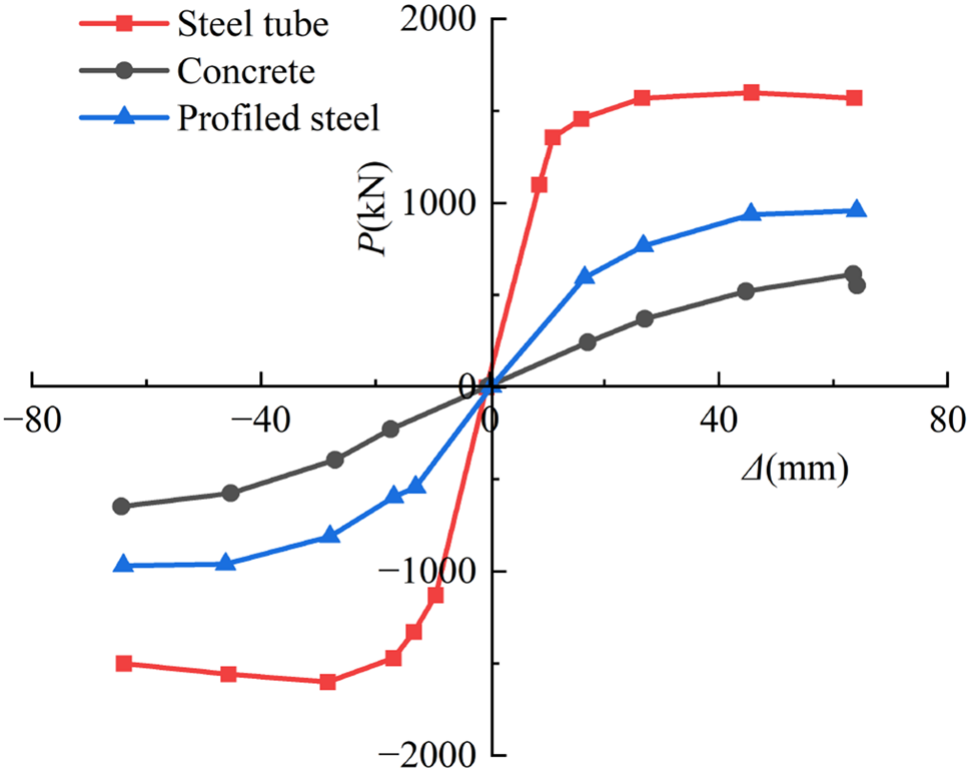
Skeleton curves of *P-*Δ relations.

The *P-*Δ hysteresis curves and skeleton lines of each component were computed for three sides exposed to fire to evaluate he level of contribution of each element to the seismic performance of SRCFST members after fire, as illustrated in Figs.19 and 20. As can be observed, steel tubes have the highest hysteresis curves, most excellent peak loads under all loading levels, stiffest elastic phases, and best energy dissipation capacities. Profiled steel comes in second place and concrete comes in last. This is since following the fire, the temperature of the steel tube periphery quickly increased and transferred to the center of the section. As a result of the natural cooling conditions, the material properties of steel have since recovered, allowing it to bear most of the reciprocal load. Concrete serves as a naturally occurring protective layer for the profiled steel, lowering section temperature, preventing early local buckling, and safeguarding the interior steel sections and periphery steel tube from harm. Additionally, since the steel tube acts as a barrier against forming oblique fissures in the concrete, this kind of component continues to perform well seismically even after a fire because of the synergistic interaction between the steel tube, profiled steel, and concrete.

## Parameter analysis of seismic performance

The ductility coefficient *μ* is employed as seismic indices for the example of three-sided fire in order to further examine the effect law of each parameter on the seismic performance of the concrete members with internal steel sections after the non-uniform fire. Parametric analysis is then carried out for each parameter in the range of parameters commonly used in engineering; the main parameters are heating time, axial pressure ratio, slenderness ratio, and steel content rate. Table 5 displays the precise values.

**Table 5:** Summary of specimen dimensions of the post-non-uniform fire seismic performance.

Parameter	values	Default values
Heating time *t*_h_/(min)	30, 60, 90, 120,	90
Axial compression ratio *n*	0.1, 0.3, 0.5, 0.8	0.5
Slenderness ratio *λ*	10, 30, 50, 70	30
profiled steel ratio *α*_s_	0.03, 0.05, 0.07, 0.09	0.05
Steel tube ratio *α*_t_	0.05, 0.08, 0.15, 0.20	0.08
Concrete cubic compressive strength *f*_cu_/(N/mm^2^)	20, 40, 60, 80	60
Yield strength of steel tube *f*_yt_/(N/mm^2^)	235, 345, 390, 420	345
Yield strength of profiled steel *f*_ys_/(N/mm^2^)	235, 345, 390, 420	345
Protective layer thickness *a/*(mm)	0, 5, 10, 15	0

### Axial compression ratio

**Figure 21:**
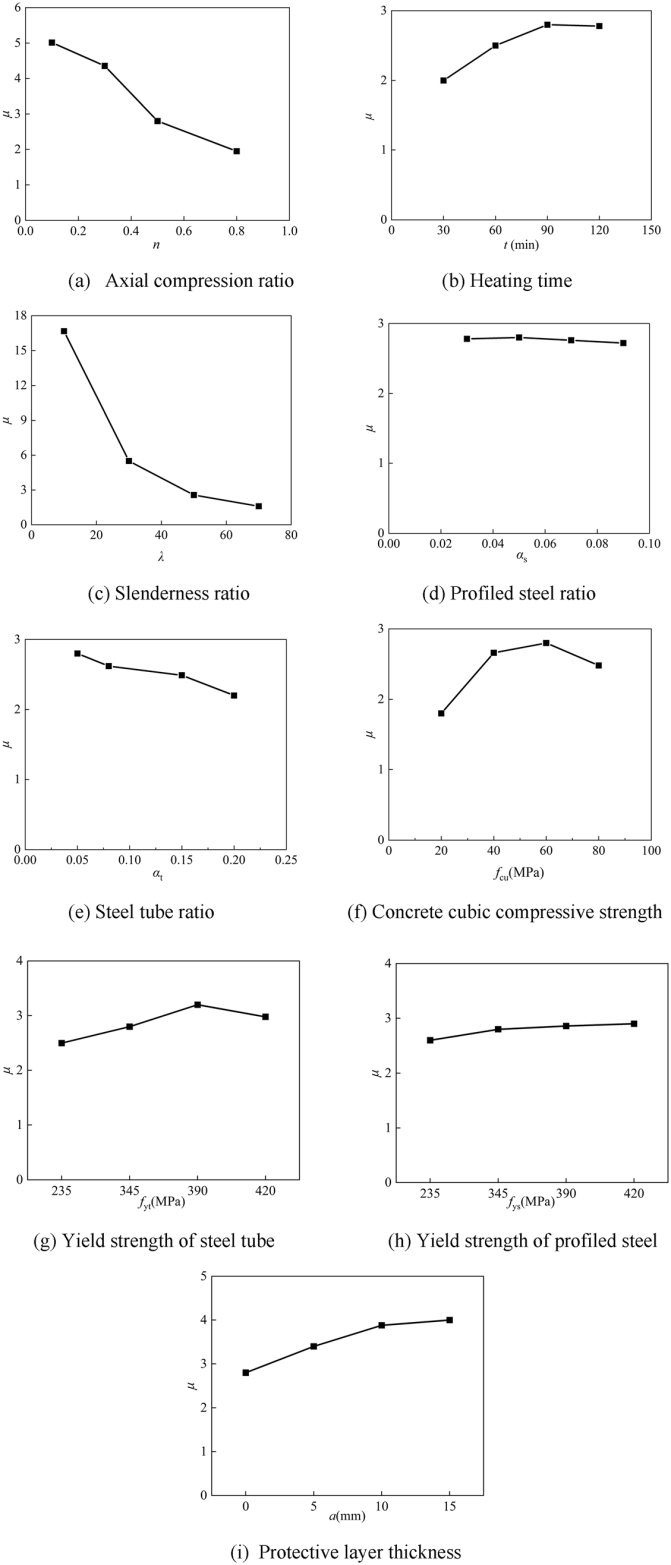
Analysis of ductility coefficient parameters.

The effect of the axial compression ratio on the three-sided ductility coefficient of the SRCFST members after a fire is shown in Fig. 21a. It is evident that the axial compression ratio has a more significant overall influence. When the axial compression ratio is 0.3, 0.5, or 0.8, compared to the axial compression ratio of 0.1, the ductility coefficients are reduced by 13.13%, 44.15%, and 61.15%, respectively. Therefore, it is essential to regulate the axial pressure ratio restrictions of the members while performing structural design.

### Heating time

When the fire duration is less than 90 min, the ductility coefficient of the specimen typically tends to increase as the fire time is extended, as shown in Fig. 21b. This is because a longer fire time causes the ultimate compressive strain of columns to grow, which causes the concrete to sustain delayed crush damage and improve the ductility. Due to the high historical maximum temperature of the member section and the severe degradation of the material characteristics, the ductility coefficient drops when the fire period is more than 90 min.

### Slenderness ratio

Figure 21c illustrates how the length to slenderness ratio affects the ductility coefficient of SRCFST components after fire on three sides. The ductility coefficient drops down dramatically as the slenderness ratio increases. The ductility coefficient is reduced by 65.4% for *λ* = 30 compared to *λ* = 10, 52.8% for *λ* = 50 compared to *λ* = 30, and 38.2% for *λ* = 70 compared to *λ* = 50. Therefore, the length ratio of column members should be reasonably selected when designing the structure to avoid premature damage to the members due to the excessive length ratio.

### Profiled steel ratio

The influence of steel content on the ductility coefficient is minimal, as shown by Fig. 21d, where the ductility coefficient tends to decline as the profiled steel ratio rises.

### Steel tube ratio

As the ratio of steel tubes increases, the ductility coefficient tends to decrease, when *α*_t_ is 0.08, 0.15, and 0.2, respectively, the ductility coefficient rose by 7.86%, 11.10%, and 21.43%, as shown in Fig. 21e. This is because the steel tube contributes most to the stiffness and bearing capacity after fire, since more steel increases stiffness while decreasing ductility.

### Concrete cubic compressive strength

The effect of concrete compressive strength on the displacement ductility factor µ is shown in Fig. 21f. It can be seen that the development of compressive strength of concrete on the ductility coefficient tends to increase first and then decrease. The ductility factor increases gradually as the compressive strength of concrete rises when *f*_cu_ is less than 60 MPa; for example, it rises by 53.7% when *f*_cu_ is between 20 and 40 MPa and by 13.8% when *f*_cu_ is between 40 and 60 MPa. The ductility coefficient declines when *f*_cu_ over 60 MPa, and 80 MPa is 15% less ductile than 60 MPa. However, the overall effect of concrete compressive strength on displacement ductility coefficient is insignificant.

### Yield strength of steel tube

As shown in Fig. 21g, when the yield limit of the steel pipe is lower than 390 MPa, the ductility coefficient increases slightly with the increase of the yield limit. The ductility coefficient starts to decline when steel pipe yield limit increases over 390 MPa. Overall, there isn’t much of an impact on the ductility coefficient due to the steel tube's yield limit.

### Yield strength of profiled steel

The relationship between the yield limit and the ductility coefficient of profiled steel is shown in Fig. 21h.The ductility improved by 7.7% for* f*_ys_ = 345 MPa compared to *f*_ys_ = 235 MPa, 2.1% for *f*_ys_ = 390 MPa compared to *f*_ys_ = 345 MPa, and 1.4% for *f*_ys_ = 420 MPa compared to *f*_ys_ = 390 MPa. It is evident that the ductility coefficient marginally increases, but only slightly, as the yield limit rises.

### Protective layer thickness

With the increase of the thickness of the protective layer, the ductility coefficient of the member after fire tends to increase. Figure 21i shows that the ductility of the member with a = 5 mm is increased by 21.43% when compared to the bare column, that of the member with a = 10 mm is increased by 14.12% when compared to that of the member with a = 5 mm, and that of the member with a = 15 mm is increased by 3.4% when compared to that of the member with a = 10 mm.The growth is progressively slowed down when the protective layer thickness is increased because, once it reaches a particular thickness, the overfire temperature of the cross-section stabilizes and the material qualities do not significantly deteriorate as a result of the fire. To guarantee the safety and dependability of the components in the project, the installation of a fire protection layer is the most straightforward method.

## Conclusions

The numerically simulated on seismic performance of steel-reinforced concrete-filled rectangular steel tubes after exposure to non-uniform fire was presented in this paper. The conclusions were obtained as follows:

- (1) Steel-reinforced concrete-filled rectangular steel tube members of the four sides of the uniform fire, the temperature field is biaxially symmetric, three sides of the fire, single-sided fire, the temperature field is uniaxially symmetric, the adjacent side of the fire, the temperature field is not symmetric. As the number of fire surfaces decreases, the overfire temperature at the center of the section decreases. Due to the inhomogeneity of the temperature field distribution, the non-uniform fire has two impacts on the mechanical properties of the members, namely increased deflection and other eccentricity. As a result, the mechanical characteristics after the fire vary from those of the uniform fire.
- (2) Increased fire surface results in decreased member carrying capacity during various fire regimes, increased stiffness degradation, decreased ductility coefficient, and reduced ability to dissipate energy.
- (3) After non-uniform fire under reciprocating load, the steel pipe bears the most significant burden, the steel section is in second place, and the concrete is in third place. However, the presence of concrete improves the stability of the steel pipe and profiled steel. It prevents premature buckling of the steel tube and profiled steel, so the components interact to make this type of member have better seismic performance after fire.
- (4) The ductility coefficient will be greatly lowered when axial pressure ratio and length to slenderness ratio grow, thus the value should be rigorously managed while constructing the structure. The most effective way to assure the safety of structural elements is to provide fire protection since the ductility coefficient of parts covered with protective layers rises significantly after a fire.

## Supplementary Information

Supplementary Information.

## Data Availability

We hereby state that all data generated or analysed during this study are included in this published article (and its Supplementary Information files).

## List of symbols

*A*_c_: Section area of concrete (m^2^)
*A*_s_: Section area of profiled steel (mm^2^)
*A*_t_: Section area of steel tube (mm^2^)
*B*: Width of the cross-section (mm)
*D*: Length of the cross-section (mm)
*L*: Length of the specimen (mm)
*E*: Energy dissipation (J)
*K*_j_: Secant stiffness
*α*_t_: Steel tube ratio
*α*_s_: Profiled steel ratio
*t*_h_: Heating time (min)
*t*_s_: Thickness of the steel tube (mm)
*f*_yt_: Yield strength of steel tube (MPa)
*f*_cu_: Concrete cubic compressive strength (MPa)
*f*_ys_: Yield strength of profiled steel (MPa)
*n*: Axial compression ratio of column
*a*: Protective layer thickness (mm)
*μ*: Ductility coefficient of column
Δ_u_: Failure displacement (mm)
Δ_y_: Yield displacement (mm)
Δ_max_: Ultimate displacement (mm)
Δ_j_: Displacement corresponding to *P*_j_ under the first cycle of level j (mm)
*P*_y_: Yield load (kN)
*P*_max_: Ultimate strength (kN)
*P*_u_: Failure load (kN)
*P*_j_: Peak load under the first cycle of level j (kN)
*e*: Load eccentricity
*N*_F_: Loads acting on members under fire (kN)
*N*_0_: Axial force applied to the column after the fire (kN)
